# Evaluation of Ethos intelligent optimization engine for left locally advanced breast cancer

**DOI:** 10.3389/fonc.2024.1399978

**Published:** 2024-07-01

**Authors:** Jessica Prunaretty, Laura Lopez, Morgane Cabaillé, Céline Bourgier, Aurélie Morel, David Azria, Pascal Fenoglietto

**Affiliations:** Radiotherapy Department, Montpellier Regional Cancer Institute, Montpellier, France

**Keywords:** ethos, IOE, automation, AI, breast cancer

## Abstract

**Purpose:**

To evaluate the feasibility to use a standard Ethos planning template to treat left-sided breast cancer with regional lymph nodes.

**Material/Methods:**

The tuning cohort of 5 patients was used to create a planning template. The validation cohort included 15 patients treated for a locally advanced left breast cancer randomly enrolled. The Ethos planning template was tuned using standard 3 partial arc VMAT and two collimator rotation configurations: 45/285/345° and 30/60/330°. Re-planning was performed automatically using the template without editing. The study was conducted with a schedule of 42.3 Gy in 18 fractions to the breast/chestwall, internal mammary chain (IMC) and regional lymph nodes (“Nodes”). The PTV was defined as a 3D extension of the CTV with a margin of 7 mm, excluding the 5mm below the skin. The manual treatment plans were performed using Eclipse treatment planning system with AAA and PO algorithms (v15.6) and a manual arc VMAT configuration and imported in Ethos TPS (v1.1) for a dose calculation with Ethos Acuros algorithm. The automated plans were compared with the manual plans using PTV and CTV coverage, homogeneity and conformity indices (HI and CN) and doses to organs at risk (OAR) via DVH metrics. For each plan, the patient quality assurance (QA) were performed using Mobius3D and gamma index. Finally, two breast radiation oncologists performed a blinded assessment of the clinical acceptability of each of the three plans (manual and automated) for each patient.

**Results:**

The manual and automated plans provided suitable treatment planning as regards dose constraints. The dosimetric comparison showed the CTV_breast D99% were significantly improved with both automated plans (p< 0,002) while PTV coverage was comparable. The doses to the organs at risk were equivalent for the three plans. Concerning treatment delivery, the Ethos-45° and Ethos-30° plans led to an increase in MUs compared to the manual plans, without affecting the beam on time. The average gamma index pass rates remained consistently above 98% regardless of the type of plan utilized. In the blinded evaluation, clinicians 1 and 2 assessed 13 out of 15 plans for Ethos 45° and 11 out of 15 plans for Ethos 30° as clinically acceptable.

**Conclusion:**

Using a standard planning template for locally advanced breast cancer, the Ethos TPS provided automated plans that were clinically acceptable and comparable in quality to manually generated plans. Automated plans also dramatically reduce workflow and operator variability.

## 1 Introduction

Breast-conserving surgery followed by whole breast irradiation (WBI) is the current standard of care for patients with early stage breast cancer (BC) (1). Although the American Society for Radiation Oncology (ASTRO) does not recommend intensity modulated radiation therapy (IMRT) for the routine delivery of WBI after breast-conserving surgery, some studies have shown that the use of IMRT is increasing worldwide (2, 3). In recent decades, IMRT has played a crucial role in improving plan quality. However, it has also introduced complexity into the treatment planning process leading to an inter-operator variability (4, 5) and an increased planning time.

The strategy of planning automation has shown promising results in standardizing treatment planning while maintaining plan quality and reducing workload (6, 7). Three methods are currently commercially available. First, knowledge-based planning (KBP) relies on knowledge from previous cases to predict an achievable dose in a new case of a similar population (8, 9). Apaza Blanco et al. (10) evaluated two knowledge-based VMAT models for breast cancer using the C-arm accelerator and demonstrated a plan quality equivalent to the planner’s experience. Esposito et al. (11) published similar results using Tomotherapy. The multicriteria optimization (MCO) is based on pareto-optimal plan proposals where one criterion cannot be improved without worsening at least one other criterion (12, 13). Finally, the template-based planning uses an iterative approach of progressive optimization that mimics the planning process by a skilled planner. This method requires the creation of a wish‐list including beam setup, dose prescriptions and planning objectives for each treatment site‐specific clinical data. The Ethos Treatment Planning System (TPS) uses the latter strategy. Ethos^®^ Therapy, marketed by Varian Medical System, includes a Halcyon^®^ linac upgraded with fully integrated and automated online ART using CBCT images and artificial intelligence (14). The Ethos TPS uses an Intelligent Optimization Engine (IOE) that automatically drives the Photon Optimizer algorithm. The IOE is designed to perform all the necessary steps to produce high quality dose distributions that match the clinical expectations for the plan, to ensure dosimetric accuracy. The performance of the IOE has been evaluated for pelvis (15–18), head and neck (19, 20), partial breast (21), and lung (22). However, to our knowledge, it has not yet been evaluated for breast cancer including regional lymph nodes. The aim of this study was to evaluate the quality of whole breast treatment plans automatically generated by the Ethos IOE using a planning directive template. The automatically generated plans will be compared to manual plans using dose metrics. A blinded assessment will be performed for a clinical approval.

## 2 Materials and methods

### 2.1 Patient description

Patients underwent a 2.5 mm slice thickness computed tomography (CT) scan (GE Optima CT580, General Electric Healthcare, Waukesha, WI, USA) in the supine position with free breathing, and with both arms above the head with personalized foam cushions.

**Figure 1:**
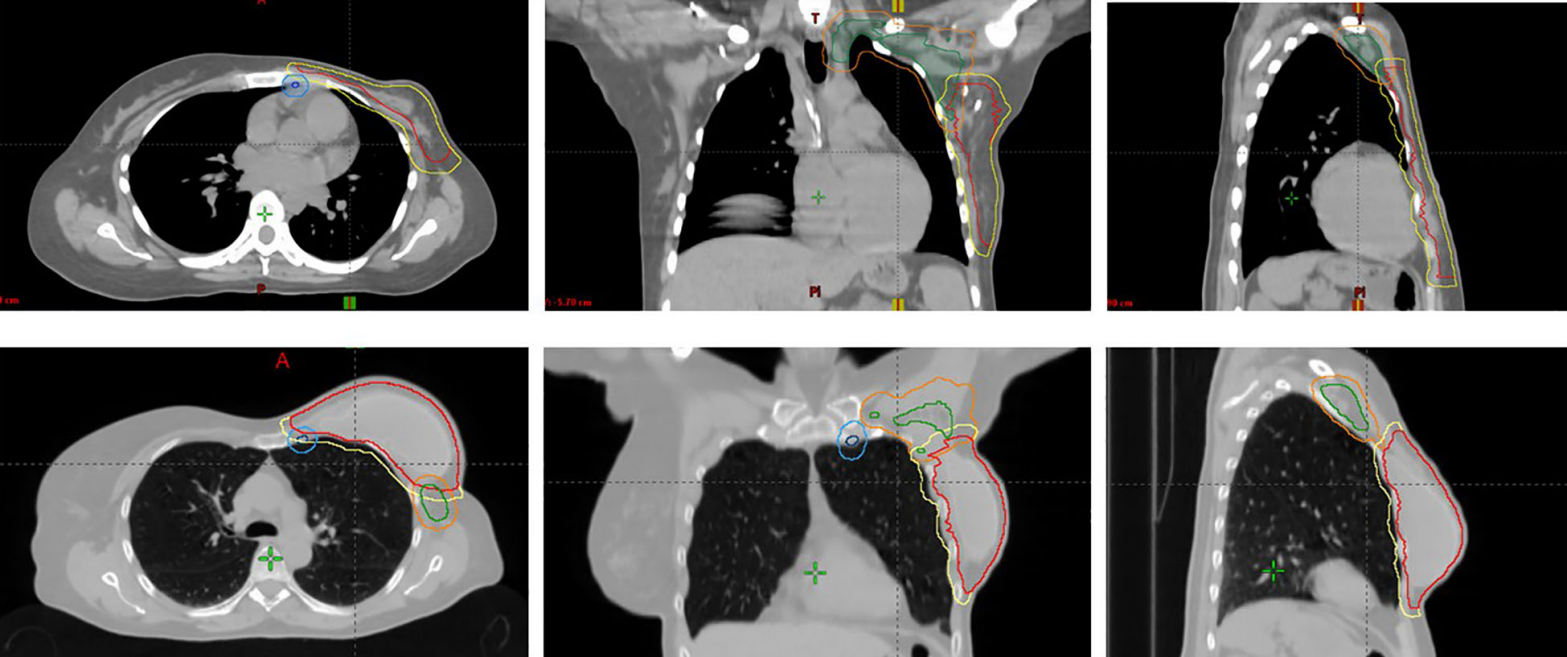
Two examples of patient CT images in axial (left), coronal (center), and sagittal (right) views showing PTV breast/chestwall (yellow), PTV_IMC (light blue), PTV_nodes (orange), CTV breast/chestwall (red), CTV_IMC (dark blue) and CTV_nodes (green).

The ESTRO consensus guidelines (23, 24) were used to delineate the target volumes, breast/wall, and axillary (Berg I), subclavicular (Berg II, III) and supraclavicular (Berg IV) lymph nodes (hereafter Nodes); and the internal mammary chain (IMC). The PTV was defined as a 3D extension of the CTV with a margin of 7 mm. All PTVs and CTVs were limited to 5 mm under the skin. Organs at risk were delineated according to the French RecoRad recommendations (25) using TheraPanacea software (26) and AW Server (General Electric). PTV volumes are listed in **Table 1** and two examples of patient CT images are shown in **Figure 1**.

**Table 1:** Volume descriptions of PTV breast, PTV_IMC and PTV_Nodes.

Patients	Volume (cc)		
	PTV_Breast	PTV_IMC	PTV_Nodes
1	619,35	37	61,38
2	762,96	45,32	294,66
3	689,64	44,43	286,88
4	890,22	47,37	355,63
5	731,91	50,4	202,73
6	1293,55	48,36	242,89
7	746,52	46,33	82,74
8	1835,12	64,69	101,78
9	1106,12	52,88	394,75
10	579,28	35,41	95,36
11	1041,43	42,17	196,68
12	588,85	43,88	276,5
13	471,76	43,33	218,38
14	1559,34	42,58	140,67
15	850,45	46,02	281,63

### 2.2 Departmental treatment planning workflow

**Figure 2:**
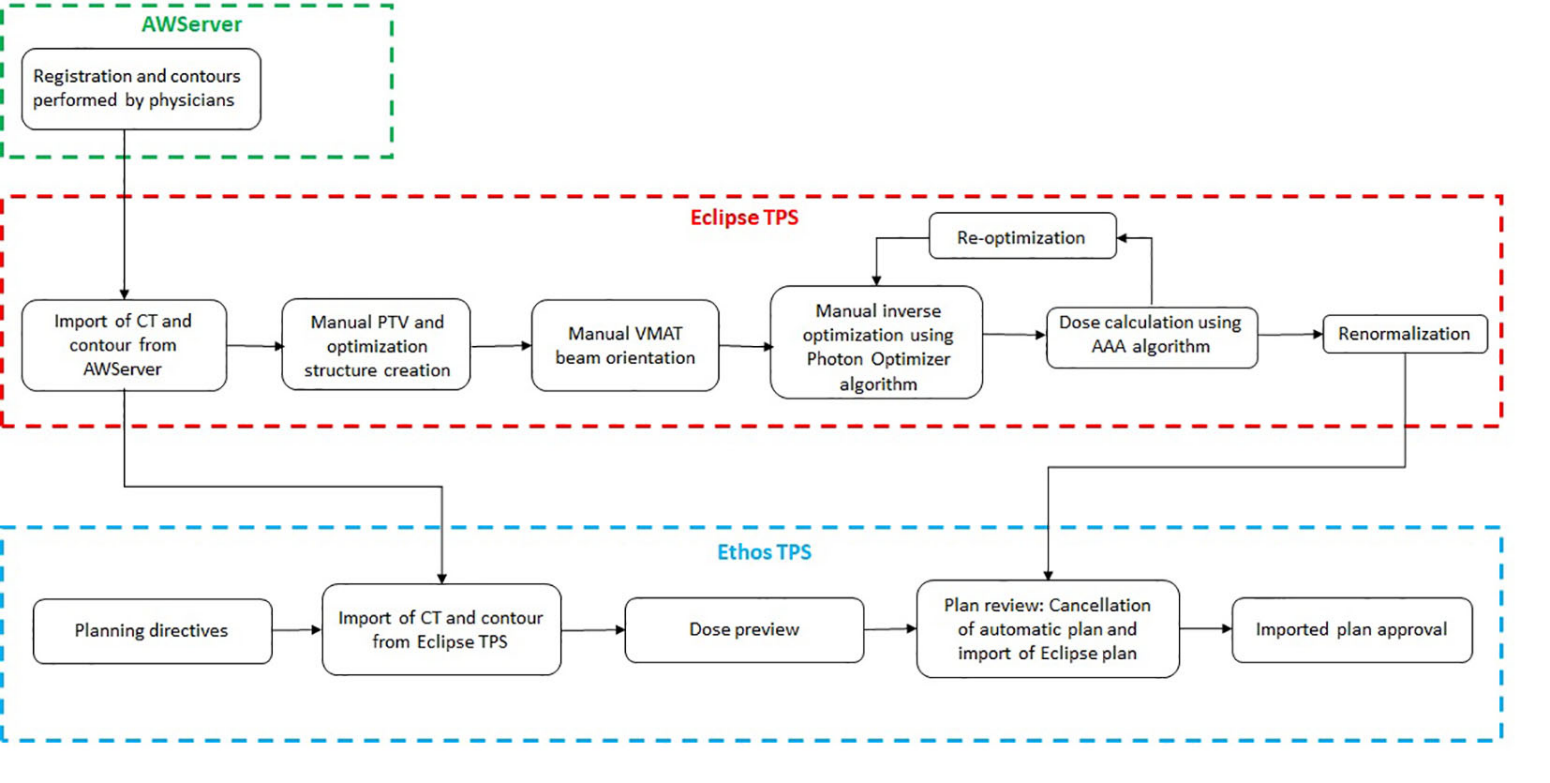
Departmental workflow for the Ethos breast treatment planning.

The Ethos treatment planning system (TPS) offers a choice of 5 fixed beam configurations: 7–9-12 IMRT fields and 2–3 full-arc VMAT (14). However, these configurations are not adapted for breast irradiation with regional nodes where partial-arc VMAT is preferred (27, 28). Therefore, a beam geometry using the Eclipse TPS is required. The current departmental workflow consists of a manual planning with the Eclipse treatment planning system (Varian, Medical Systems, Palo Alto, CA, USA) using the Photon Optimizer and AAA algorithms (PO, AAA, v15.6, Varian, Medical Systems, Palo Alto, CA, USA). Once the treatment plan is clinically acceptable on the Eclipse TPS, it is then imported into the Ethos TPS (v1.1) and calculated with the Ethos Acuros algorithm for clinical approval. Renormalization of the plan was necessary to compensate for the dose differences between the two algorithms (29, 30). The departmental workflow is detailed in **Figure 2**.

### 2.3 Manual treatment planning

The study was conducted with a schedule of 42.3 Gy in 18 fractions to the breast/chestwall, internal mammary chain (IMC) and regional lymph nodes (“Nodes”) according to the HYPOG-01 clinical trial (6). The dose constraints for CTVs and organs at risk (OAR) are shown in **Table 2**. No PTV dose constraints are defined in this protocol. Patients were treated with the Ethos linear accelerator using Volumetric Modulated Arctherapy (VMAT) technique. The arc amplitude was 240° (from 179° to 300°, and vice versa). Treatment descriptions are detailed in **Table 3**. The number of arcs and collimator angles are defined according to the operator and the anatomical complexity of the patient. The manual plans will be referred to as “Eclipse” throughout the rest of the study.

**Table 2:** Dose constraints for CTVs and organs at risk.

CTV constraints		
CTV Boost	D_95%_ ≥ 49.6Gy	D_2%_ ≤ 56.4Gy
CTV Breast/Chestwall	D_95%_ ≥ 40.2Gy	D_2%_ ≤ 45.7Gy
CTV nodes (IMC and CLN)	D_95%_ ≥ 40.2Gy	D_2%_ ≤ 44.8Gy
OAR constraints		
Heart	V_17Gy_< 10%	V_35Gy_< 5%
Ipsilateral lung	V_17Gy_< 30%	D_mean_< 16Gy
Lungs	V_17Gy_< 22%	
Brachial plexus	D_max_< 46.25Gy	
Spinal cord	D_max_< 38.54Gy	
Contralateral breast	D_mean_< 2Gy	
LAD coronary	D_max_< 17Gy (if possible)	

**Table 3:** Treatment description including VMAT geometry for each patient.

Patients	VMAT geometry	
	Number of arcs	Collimator rotation (°)
1	3	30/330/60
2	4	293/338/23/68
3	3	10/350/15
4	4	293/338/23/68
5	3	285/345/45
6	3	10/345/20
7	3	285/345/45
8	3	285/345/45
9	3	285/345/45
10	4	285/345/45/15
11	3	285/345/45
12	3	285/345/45
13	4	281/326/11/56
14	3	285/345/45
15	4	5/20/345/340

### 2.4 Automated treatment planning

An Ethos optimization template was tuned using standard 3 partial arc volumetric modulated arc therapy and two collimator rotation configurations: 45/285/345° (referred to as Ethos-45°) and 30/60/330° (referred to as Ethos-30°). The collimator rotation configurations were selected based on the clinical practice. Five patients previously treated with Ethos were randomly selected for the tuning cohort. The tuning cohort was used to create a template for planning guidelines. Clinical experience from the other centers (19, 21) and iterative planning were adapted to achieve a standard template and ensure dosimetric accuracy. Finally, fifteen patients were automatically replanned using the template without editing.

### 2.5 Plan comparison

All the treatment plans were transferred to Eclipse TPS for the purpose of conducting a side-by-side comparison. Dose metrics were compared between the three plans using some dose constraints provided by the HYPOG-01 protocol and some additional relevant parameters. Target volume coverage was assessed using more demanding parameters than those used in the clinical protocol according to our clinical practice, i.e the doses received at 99% and 95% of the volume (D_99%_ D_95%_) for the CTVs and PTVs (breast, IMC and nodes), respectively. For the ipsilateral lung, the mean dose (D_mean_) and the volume receiving 17Gy (V17Gy) were calculated. Mean doses to the heart, the contralateral lung and the right breast and maximum doses (D_max_) to the brachial plexus and LAD coronary arteries were also recorded. The homogeneity index within the whole PTV is defined by the following formula (31):


$$HI=\frac{D_{2\%}-D_{98\%}}{D_{50\%}}$$

The dose conformity was evaluated using the conformity index (CI) defined as (32):


$$CI=\;\frac{{\left(V_{95\%\left(PTV\right)}\right)}^{2}}{V_{PTV}\times V_{95\%\left(Body\right)}}$$

Where V_95%(PTV)_ and V_95(Body)_ are the volumes receiving at least 95% of the prescribed dose for the whole PTV (breast, IMC and Nodes) and body, respectively. V_PTV_ is the volume of the whole PTV. The total number of monitor units (MU) was reported for each plan. Finally, the optimization and calculation times for Ethos plans were extracted from the treatment report. Due to the retrospective nature of the study, we were unable to collect these values for Eclipse plans.

### 2.6 Quality assurance

Ethos includes Mobius3D (version 3.1, Varian Medical System), an integrated and independent quality assurance (QA) tool for dose calculation using an independent collapsed cone convolution algorithm. Pre-treatment QA was performed for each plan. The assessment metric was the global gamma pass rate with a 3%/3mm criterion and a 10% threshold. In addition, the beam-on time was estimated for each plan using Mobius3D.

### 2.7 Physician review

Two radiation oncologists specializing in breast cases thoroughly reviewed the Ethos and Eclipse plans. They performed a blinded assessment of the clinical acceptability of each of the two automated plans for each patient. During plan review, the physicians made binary decisions regarding the clinical acceptability of the plan. The Ethos optimization template was not shown to the clinicians prior to the assessment to avoid decision bias. The clinicians then blindly selected the best plan from the three proposed plans (Eclipse, Ethos-45°, Ethos-30°).

### 2.8 Statistical analyses

The Wilcoxon signed rank test was used to determine the significant difference between the Eclipse and Ethos plan metrics. A Bonferroni correction was applied and the significance level was set at 0.003.

## 3 Results

This study evaluated the viability of employing an Ethos standard planning template for treating left-sided breast cancer involving regional lymph nodes. **Table 4** provides a summary of the structures and objectives utilized, along with their corresponding priorities for the standard template. The primary objective was to control the hotspot, set at 107% of the prescribed dose. Subsequently, the template emphasized avoidance of the ipsilateral lung, coverage of the clinical target volume (CTV), protection of the heart, and coverage of the planning target volume (PTV). Lower priority was assigned to contralateral organs, with no specific avoidance strategy implemented.

**Table 4:** Summary of the objectives used in the standard template.

Priority	Structure	Objectives
1	Body	D_max_< 45Gy
	Left lung	V_16.5Gy<_ 20%
		V_25Gy_< 10%
	CTV_Breast	D_99%_ > 95%
	CTV_IMC	D_99%_ > 97%
	CTV_Nodes	D_99%_ > 97%
	Heart	D_mean_< 7Gy
	PTV_Breast	D_95%_ > 95%
	PTV_IMC	D_95%_ > 95%
	PTV_Nodes	D_95%_ > 95%
	PTV_IMC	D_0.1cc_ ≤ 105%
2	Left lung	D_mean_< 10.8Gy
	Right lung	D_mean_< 4.2Gy
	Right breast	D_mean_< 4.2Gy
3	Left lung	V_4Gy_< 80%
	Heart	D_5%_ ≤ 25Gy
	Spinal cord	D_max_< 25Gy
	Spinal cord + 10mm	D_max_< 33Gy

**Figure 3:**
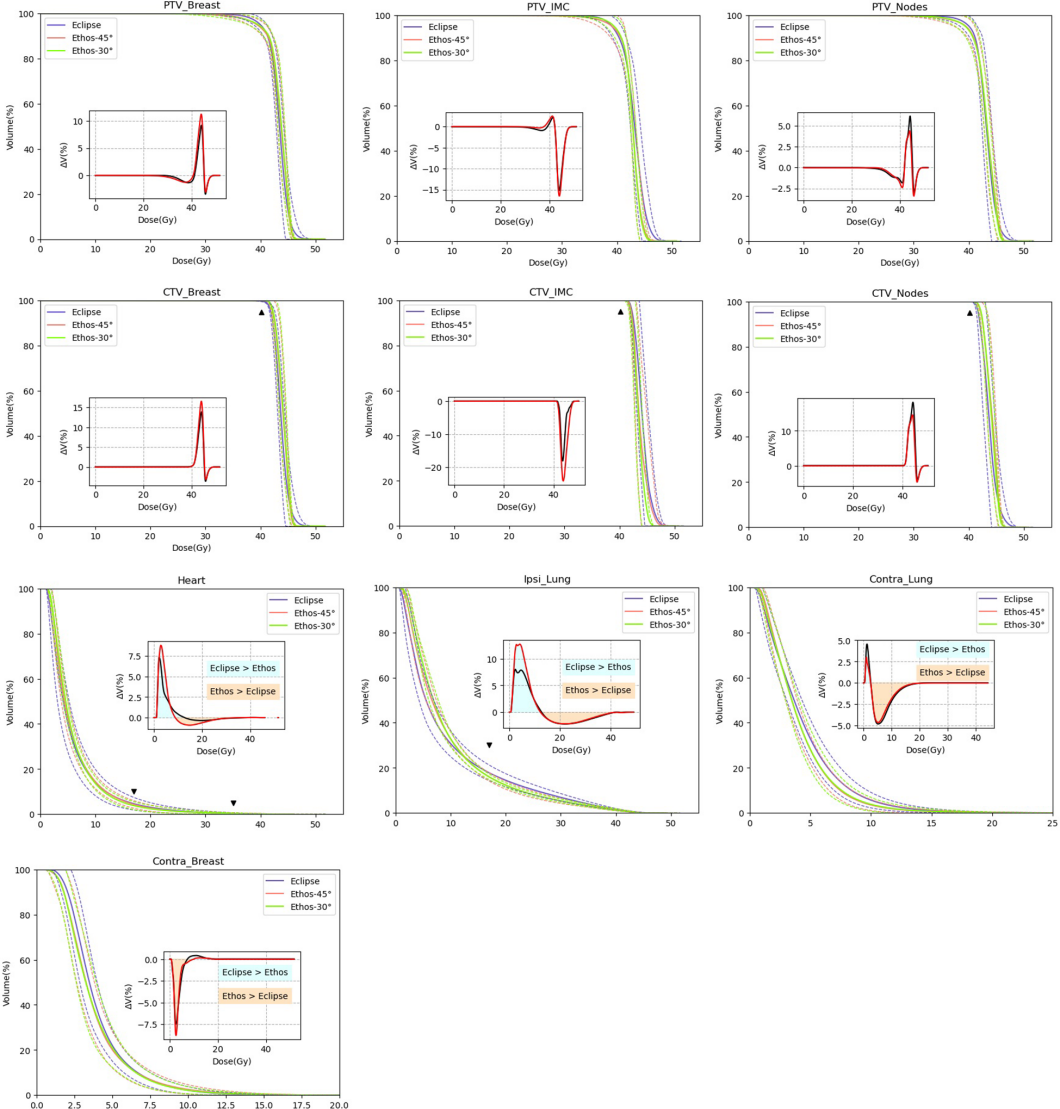
Comparison of mean DVH (solid line) of Eclipse (blue) and Ethos automatic plan (red and green for Ethos-45° and Ethos-30°, respectively). Dashed lines show the standard deviation. The triangle figures show the HYPOG constraints. Insets show the mean of DVH difference between Ethos-45° minus Eclipse (black) and Ethos-30° minus Eclipse (red).

The manual and automated plans provided suitable treatment planning in terms of HYPOG-01 dose constraints (**Figure 3** and **Table 5**). PTV coverage was similar between the Eclipse (manual) and both Ethos (automated) plans. The conformity and homogeneity indices were not statistically different between the three plans. Only the CTV_breast coverage were significantly improved with both Ethos plans (p< 0,002). The mean dose to the organs at risk was equivalent between the three plans. However, the dose distributions were different. For protection of the heart and both lungs, ΔDVH showed that the Eclipse plans delivered fewer low doses than the Ethos plans while the Ethos plan provided better protection at intermediate and high doses (**Figure 2**). For the contralateral breast, however, the trend was reversed.

**Table 5:** Dose metric results (mean ± standard deviation) for PTVs and organs at risk (OAR) for Eclipse, Ethos-45° and Ethos-30° treatment plans.

		Eclipse	Ethos-45°	Ethos-30°
PTV_Breast	D_95%_	39,91 ± 1,41	39,48 ± 1,58	39,45 ± 2,02
PTV_IMC		39,42 ± 1,61	39,48 ± 2,15	39,76 ± 1,47
PTV_Nodes		40,17 ± 1,78	39,32 ± 2,02	39,52 ± 1,73
HI		0,18 ± 0,05	0,2 ± 0,04	0,2 ± 0,06
CI		0,83 ± 0,03	0,84 ± 0,04	0,85 ± 0,04
CTV_Breast	D_99%_	41,11 ± 0,32	41,82 ± 0,32	41,89 ± 0,36
CTV_IMC		42,83 ± 1,14	42,18 ± 0,60	41,96 ± 0,49
CTV_Nodes		41,94 ± 0,92	41,93 ± 0,64	41,99 ± 0,44
Heart	D_mean_	5,98 ± 1,15	6,1 ± 0,52	6,03 ± 0,50
Ipsi_Lung	D_mean_	9,62 ± 1,16	9,77 ± 0,60	10,00 ± 0,50
	V_17Gy_	17,79 ± 3,44	15,97 ± 1,85	15,99 ± 1,23
Contra_Lung	D_mean_	4,37 ± 0,62	4,09 ± 0,50	4,10 ± 0,64
Contra_Breast	D_mean_	4,01 ± 0,55	3,83 ± 0,61	3,81 ± 0,65
LAD coronary	D_max_	23,96 ± 10,68	22,92 ± 9,45	24,67 ± 9,47
Brachial plexus	D_max_	45,19 ± 1,16	45,28 ± 1,40	45,44 ± 1,12

Regarding the treatment delivery, the Ethos-45° and Ethos-30° plans resulted in MU increases of 15.5 and 17.3%, respectively, compared to the Eclipse plans, with no impact on the beam on time (**Table 6**). Pre-treatment verification with mobius3D showed that mean gamma index pass rates remained above 98% regardless of the plans used. Ethos plan generation (including optimization and dose calculation) was in the range of 14 to 15 minutes.

**Table 6:** Mean [Min-Max] of total MUs, gamma passing rates, plan generation time and beam on time for Eclipse, Ethos-45° and Ethos-30° treatment plans.

	MU	Gamma passing rate (3%3mm)	Optimization + Calculation time (s)	Beam on time (s)
Eclipse	827,6 [744,4 - 987,7]	98,41 [97,4 - 99,3]		167 [135 -208]
Ethos-45°	955,6 [819,2 - 1149,7]	98,77 [97,8 - 99,4]	911 [730 - 1139]	166 [148 - 187]
Ethos-30°	971,0 [837,6 - 1081,3]	98,91 [98,3 - 99,2]	857 [658 - 1154]	169 [158 - 179]

**Figure 4:**
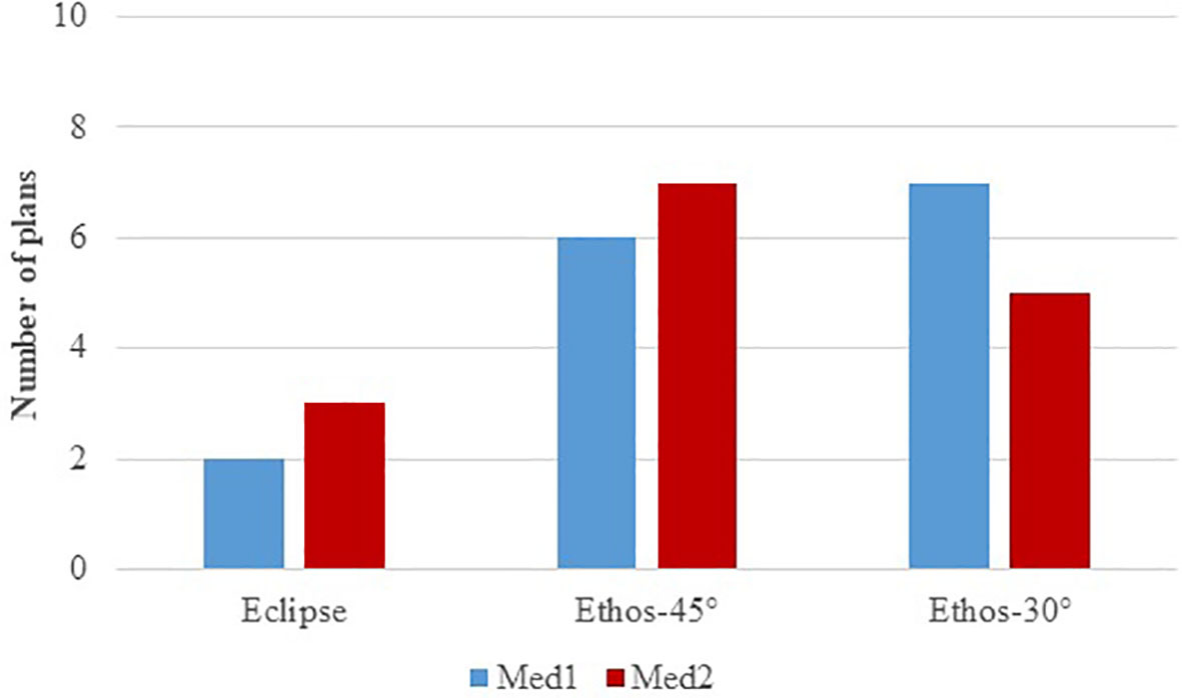
Physicians’ choice of treatment plan during the blinded comparison between manual (Eclipse) and automatic (Ethos-45° and Ethos-30°) plans.

In the blinded assessment, both clinicians 1 and 2 found 13/15 and 11/15 of the plans clinically acceptable for Ethos 45° and Ethos 30°, respectively. Furthermore, if an Ethos plan was not clinically acceptable, the second Ethos geometry was acceptable. Finally, **Figure 4** shows the physician’s choice of treatment plan in the blinded comparison between manual (Eclipse) and automatic (Ethos-45° and Ethos-30°) plans. Physician 1 selected 86.7% of the Ethos plans (Ethos-45° and Ethos-30°combined) while the physician 2 selected 80% of the Ethos plans.

## 4 Discussion

This study presents the first investigation of Ethos treatment planning for left-sided locally advanced breast cancer. We conduct a comprehensive assessment, both quantitative and qualitative, of treatment plans generated automatically.

The automated plans provided an adequate treatment plan with respect to the HYPOG-01 dose constraints, and the quality of the plans was similar to the manual plans. These results are consistent with the literature for other disease sites (16, 21, 22). Furthermore, a difference in dose distribution between low and high dose was observed between manual and automated treatment plans. Pogue et al. (22) developed an Ethos treatment plan template for locally advanced lung cancer and reported similar results for the organs at risk. In their study, they compared the initial treatment plan using C-arm accelerators with the Ethos treatment plans. Due to the different multi-leaf collimator (MLC) design between the accelerators, they could not determine how much of the difference in the Ethos plan was due to the Ethos double-banked MLC and how much was due to the IOE. In contrast, our study was performed with the Ethos accelerator only. Therefore, the dosimetric differences observed cannot be attributed to the MLC.

In addition, blinded physician review showed that at least 73% of the automated plans were clinically acceptable without edits, demonstrating the robustness of the standard template.

In terms of planning and treatment efficiency, automated treatment planning resulted in an increase in MUs, consistent with findings from another automated planning engine (16, 20). This suggests an increase in plan complexity, but without any impact on the quality assurance.

In addition to the high plan quality achieved with the automated treatment plan, the interest of this work was to reduce the workload caused by switching back and forth between two TPSs. Although the time savings have not been fully quantified, it is reasonable to assume that plan preparation time will be reduced. In our clinical experience, the time required for a planner to manually generate a clinically acceptable breast VMAT plan ranged from 60 to 180 minutes, depending on anatomical complexity. The two Ethos plans optimized for each patient took 30 minutes without intervention. Note that the time required to select the planning directive and planning image, and then manually associate the structure set, remains unchanged between the two workflows.

There was no significant difference in dose metrics between the two collimator rotation configurations for the automated plans. However, in some cases where an Ethos plan was clinically unacceptable in the blinded assessment, the second Ethos geometry was acceptable. The interest of the two geometries is to propose two different plans without editing the planning template.

Finally, we have chosen to maintain this VMAT geometry despite several studies reporting that the Ethos optimization time is significantly longer with VMAT than with IMRT (16, 17). The two main reasons for this are our extensive experience with breast VMAT (33–36) and the fact that all our patients are treated with Ethos in the IGRT mode for breast cancer: the planning time is affected but not the length of the session. In the context of an adaptive session with a daily re-optimization, an IMRT beam geometry should be considered in order not to increase session times (37). In addition, only a single isocenter was examined in this study. Due to the limited maximum field size of the Ethos (28 x 28 cm²), the larger whole breast with regional nodes could not be covered by a mono-isocenter technique. In this case, the standard planning template with a specific multi-isocenter technique should be investigated.

Future research will encompass the clinical integration and prospective application of this standard template. Moreover, the methodology outlined in this study will be employed for other treatment protocols (such as simultaneous integrated boost for breast cancer) and extended to other disease sites.

## 5 Conclusion

This study demonstrated the feasibility of the Ethos Intelligent Optimization Engine to generate high quality automated plans using a standard planning template for left-sided locally advanced breast cancer. Planning automation reduces the need for human intervention, thereby reducing both the workload and operator variability.

## Data availability statement

The original contributions presented in the study are included in the article/supplementary material. Further inquiries can be directed to the corresponding author.

## Ethics statement

Ethical approval was not required for the study involving humans in accordance with the local legislation and institutional requirements. Written informed consent to participate in this study was not required from the participants or the participants’ legal guardians/next of kin in accordance with the national legislation and the institutional requirements.

## Author contributions

JP: Conceptualization, Investigation, Methodology, Supervision, Writing – original draft. LL: Investigation, Writing – review & editing. MC: Investigation, Writing – review & editing. CB: Writing – review & editing. AM: Writing – review & editing. DA: Writing – review & editing. PF: Conceptualization, Methodology, Writing – review & editing.
